# It’s not what you think: shaping beliefs about a robot to influence a teleoperator’s expectations and behavior

**DOI:** 10.3389/frobt.2023.1271337

**Published:** 2023-12-21

**Authors:** Daniel J. Rea, James E. Young

**Affiliations:** ^1^ Faculty of Computer Science, University of New Brunswick, Fredericton, Canada; ^2^ Department of Computer Science, University of Manitoba, Winnipeg, Canada

**Keywords:** teleoperation, human-robot interaction, user experience, priming, perception

## Abstract

In this paper we present a novel design approach for shaping a teleoperator’s expectations and behaviors when teleoperating a robot. Just as how people may drive a car differently based on their expectations of it (e.g., the brakes may be poor), we assert that teleoperators may likewise operate a robot differently based on expectations of robot capability and robustness. We present 3 novel interaction designs that proactively shape teleoperator perceptions, and the results from formal studies that demonstrate that these techniques do indeed shape operator perceptions, and in some cases, measures of driving behavior such as changes in collisions. Our methods shape operator perceptions of a robot’s speed, weight, or overall safety, designed to encourage them to drive more safely. This approach shows promise as an avenue for improving teleoperator effectiveness without requiring changes to a robot, novel sensors, algorithms, or other functionality.

## 1 Introduction

**FIGURE 1:**
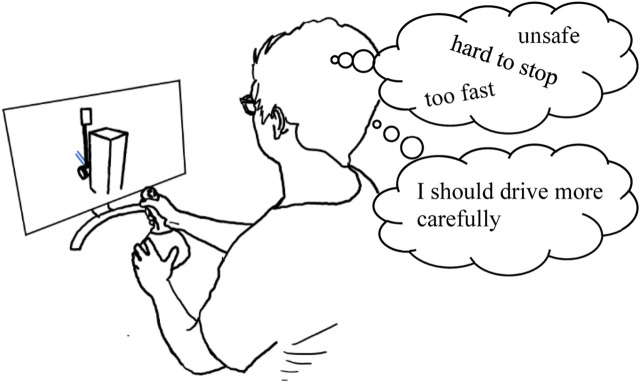
We investigate how priming an operator’s expectations of robot capabilities impacts their driving behavior and perceptions of the robot. Our study results found that priming impacted operator perception of the robot in all cases, and in some cases could affect driving safety.

Teleoperation is the act of controlling robots remotely, enabling people to explore a distant country, inspect industrial environments, or support urban-search-and-rescue, all without being physically present. This remote-control problem requires the operator to maintain awareness of the remote robot and its surrounding environment, while articulating robot commands for navigation and interaction, all in real time. This is a highly challenging task, with human error (e.g., *critical incidents–*collisions with people or the environment) remaining a major cause of operation faults (Williams, 2004; Giese et al., 2013). We propose a novel approach to this problem: exploring how to reduce human error by designing the robot or interface to shape an operator’s expectations about the robot. For example, we may lead an operator to believe a robot is dangerous or fragile, with the aim of encouraging them to drive less aggressively (e.g., as in Figure 1).

Our exploration focuses on the common challenge of navigating a robot in a remote space, where operators must explore and move about while avoiding collisions. We know from automobile driving that we can expect people to drive more safely if they anticipate dangerous conditions (Fuller, 2005) such as bumpy or icy roads, or if they suspect a car may stall or has weak brakes. Inversely, a person may drive a safer car less carefully as they rely on the safety features to manage mistakes [e.g., as with ABS brakes (Jonah et al., 2001)]. In all these examples, we note that it is the perception of safety and risk that shapes driving, even if the perceptions are not substantiated (e.g., a road may not actually be slippery). We draw inspiration from this observation and propose to develop methods for explicitly designing interfaces, the robots themselves, or how it is presented, to shape teleoperator perceptions of a robot, and thus their driving behavior.

We approach teleoperation from the perspective of priming, where we employ a range of stimuli to encourage people to recall past experiences and understanding of the world to influence their thoughts and behavior (Bargh et al., 1996; Dijksterhuis and Bargh, 2001; Doyen et al., 2012). Drawing from the automobile comparison above, we explore methods to encourage operators to perceive a robot as if it was dangerous or difficult to operate, and if this shift in perception will in turn encourage the operator to drive the robot more carefully (Figure 1). We designed two novel approaches for priming operators, aiming to shape their expectations and beliefs about the safety of their robot: using joystick stiffness (resistance to movement) to represent robot power, and using leading robot description such as calling it weak or powerful to represent general safety. Following, we conducted two studies (one per method, 49 participants total) to investigate the impact of these methods. Our results demonstrate the effectiveness of our approaches for shaping operator perceptions and expectations of the robot, including the robot’s speed, durability, and controllability; in some cases, our method resulted in changes in driving behavior and performance. These were previously reported in (Rea and Young, 2018).

In analyzing the results from our first two studies, we identified additional limitations and potential confounds relating to priming that may invalidate our previous results. Specifically, we noted that modifying joystick stiffness may impact usability (e.g., a loose joystick may be hard to control, or a heavy joystick may take effort to push) while simultaneously priming an operator on robot capability. As such, we present a follow-up study that specifically investigates this usability concern, teasing out the impact of priming. However, in this experiment we faced a new unexpected challenge: how to construct an experimental design without any priming element, to compare against our priming. That is, we had to consider: is it even possible to create and present an interface without, even inadvertently, priming an operator on robot capability? In analyzing the results of this third study, and conducting a reflective analysis of the whole body of work, we reconsider our priming work overall and the use of priming in teleoperation through a more nuanced lens. This results in a discussion and recommendations for how to consider priming in any interface work.

Overall, our results highlight the potential power of using priming as a design tool to shape operator perceptions of their robot, and in some cases, their driving behavior. Further, we present list of considerations to guide researchers in exploring priming in future teleoperation research and development. Priming is a new powerful tool for impacting teleoperation interfaces that does not require any changes to a robot’s physical capabilities, and can be used when changes to an actual robot or system would be difficult or prohibitively expensive.

## 2 Background: priming

In psychology, the term priming is used across a range of applications and methods that use a stimulus (the priming) to cause an impact on an event or interaction. In our work, we focus on behavioral priming, where exposing a person to a stimulus or concept elicits some associated knowledge from previous experience, and impacts their behavior based on that experience (Bargh et al., 1996; Dijksterhuis and Bargh, 2001; Doyen et al., 2012). For example, showing people a picture of a library can make them unconsciously speak more quietly (Henk Aarts and Dijksterhuis, 2003). In this case, people associate the stimulus (an image of a library) with their prior experience of libraries requiring quiet and change their behavior to align with that experience (e.g., speaking more softly).

Priming is broadly studied in domains outside of psychology. For example, in marketing it has been shown that priming stimuli embedded in surroundings can change evaluations of a company’s brand (Yi, 1990), and priming stimuli combined with different prior knowledge was found to change price evaluations of products (Herr, 2012). Biology has studied potential biological underpinnings of priming in order to better understand the human brain (Iacoboni, 2009). How priming may work, and its interactions with other variables is still an active area of research. However, it is clear that priming has the potential to change perceptions and behavior. Thus, we examine priming as a means of shaping teleoperation.

### 2.1 Priming methods

A broad range of priming methods have been shown to be effective in altering behavior. Various modalities have been explored such as sound–playing musical chords in the background can change the emotions written words can convey (Sollberger et al., 2003)–tangible methods such as the weight or rigidity of someone’s clipboard being used to prime perceptions of their social rank or personality (Ackerman et al., 2010)–or visual stimuli such as making people speak more quietly by simply having a picture of a library in view (Henk Aarts and Dijksterhuis, 2003). Explorations of priming effects have investigated the range in stimuli subtlety or frequency (Forster and Davis, 1984; MacLeod, 1989) showing that priming stimuli can range from rare, unnoticed stimuli, such as omitted types of words in a word list (Bargh et al., 1996) priming people to think of that word, to explicit priming attempts where the primed person is made aware of the priming attempt and affect (Jim Cheesman and PhilipMerikle, 1984; Doyen et al., 2012). While differing in size of the effect, priming can change people’s behavior in all these cases.

Priming has commonly been studied in the context of impacting social relations between two people. For example, having one person directly describe another as “mean” or “kind” can increase the likelihood that the primed characteristics will be observed (Kelley, 1950). Opinions of others can also be primed using physical props, such as seeing someone as more important when they are holding a heavier clipboard (Ackerman et al., 2010). Priming can be quite nuanced, for example, in the above example people assume the other is more difficult to interact with if the clipboard is rough (Ackerman et al., 2010).

Although one may associate priming with stimuli given only prior to an interaction, priming stimuli can also be presented frequently or continuously throughout interactions (Bargh et al., 1996; Ackerman et al., 2010). For example, driving a car with a loud engine provides ongoing priming, a constant reminder, of the car’s power. In the earlier example of the library picture influencing people to speak more quietly, the picture was present throughout the experiment (Henk Aarts and Dijksterhuis, 2003). Considering when and how often priming methods are applied should be considered in intentional priming designs.

This breadth of techniques highlights the range of potential priming methods available to teleoperation designers, and further, suggests what kinds of impacts on operators designers may expect from the use of priming methods.

### 2.2 Priming effects

Priming is often studied for its short-term effects, but priming can have long-term results, sometimes lasting for hours, weeks, or months (Sloman et al., 1988; Becker et al., 1997). After priming exposure, however, the strength of priming effects tend to weaken (Sloman et al., 1988). Repetition of the priming can counteract the weakening effect, but this may not work for all stimuli (Forster and Davis, 1984).

Priming effects can be highly context sensitive (MacLeod, 1989; Bargh et al., 1996; Henk Aarts and Dijksterhuis, 2003; Doyen et al., 2012), where the context or environment itself can be intentionally designed to prime (Henk Aarts and Dijksterhuis, 2003; Ackerman et al., 2010). For example, priming effects can vary due to the environment–such as background sounds having different impacts dependent on context (Sollberger et al., 2003)–or nuances of the task description (MacLeod, 1989).

Thus, priming is extremely diverse and nuanced, including a broad variety of methods that can be used to prime, and a similarly broad range of potential effects of the priming. As such, designing priming methods, developing expectations of the effects, and even measuring the causes and effects of priming, remains a difficult and unsolved problem (Doyen et al., 2012; Westlund et al., 2016).

### 2.3 Summary

Priming, in the context of our work, is the use of stimuli that evokes feelings or memories that can affect a person’s thoughts or behaviors. The stimuli can be given before or during interaction, may be continuous, and may be done in secret, unbeknownst to the person, or explicitly, known to the person. From this body of work, we can also imagine that priming can even be unintentional, for example, an interface design may have features, even if unintended, that influences an operator by drawing from their prior experiences, resulting in them driving more aggressively. Thus, we argue it is important for the field of human-robot interaction to understand priming broadly, and that there is a need to develop tools and frameworks to support teleoperation designers to make informed interface design decisions to better control the user experience and how those design choices impact operator perceptions and actions.

We note that the effectiveness of priming is still debated, and the science is still unclear on the limits and applications of priming (Doyen et al., 2012). Thus, this paper provides important data points, building on the work of behavioral priming in psychology, by establishing and exploring the use of priming for shaping teleoperator perception and behavior.

## 3 Related work

A core goal of research in teleoperation aims to improve operator performance, including faster task completion time, fewer critical incidents such as collisions, and lower perceived workload (Steinfeld et al., 2006; Jessie et al., 2007). Many broad approaches have been adopted, including developing novel control methods to reduce task completion time or collisions (Leeper et al., 2012; Rea et al., 2017a), supporting operator awareness of the remote area (Curtis et al., 2007; Singh et al., 2013; Endsley, 2016; Seo et al., 2017), and mental resource management to improve overall operator performance (Jessie et al., 2011; Hacinecipoglu et al., 2013; Rea et al., 2017b; Rea, 2020). These works aim to improve teleoperator performance by improving the usability of operator controls or supporting an operator’s ability to understand and correctly react to a situation. Our work is complementary to this method, where instead of developing actual new interfaces or robotic technologies, we use priming to impact teleoperator performance and perceptions by modifying their perceptions and expectations.

Priming has been broadly studied in human-computer interaction. For example, using priming in virtual reality to explore and change how people act and perceive themselves in a virtual space (Banakou et al., 2013), such as by having participants read materials prior to entering a virtual environment or to shape their experience in the space (Nunez and Blake, 2003). Other examples include the use of subliminal priming to aid learning (Chalfoun and Frasson, 2011), priming to aid performance in visual search tasks (Young et al., 2003; Harrison et al., 2013), or analyses of how experimental design choices can prime participants and impact results (Bradley et al., 2015). In much of this work, the focus is on the flexibility offered by technology (e.g., virtual interfaces) to have freedom over nuances of priming; this principle similarly applies to interfaces for teleoperating robots.

Relating to this, research has leveraged psychology to design interfaces to influence behavior. For example, using knowledge of attention and perception to increase the saliency of potential points of interest during teleoperation (Chung et al., 2013; Rea et al., 2017b), and the addition of haptic reminders have helped users notice changes in on-screen displays (Young et al., 2003). Others have used video-game inspired techniques to improve engagement or motivation to use software (Antin and Churchill, 2011; Li et al., 2012; Hamari et al., 2014), such as for the inclusion of scores and audio-visual rewards in software tutorials (Li et al., 2012). We follow this line of research by exploring the use of priming for teleoperator perceptions of a robot’s capabilities and observing how this priming may affect operator perception of the robot and behavior.

In the related study of motor-vehicle driving, research has demonstrated the importance of the driver’s perceptions and mental state: a driver’s perception of a vehicle’s capabilities and its surroundings can change automobile driving behavior (Michon, 1985; Groeger and Rothengatter, 1998; Groeger, 2002) and actual operation safety (Jessie et al., 2007). These perceptions can be shaped by haptic accelerator pedals (McIlroy et al., 2017), transmission choice (Blommer et al., 2017), vehicle type (Eyssartier et al., 2017), or even by changing people’s mood (Precht et al., 2017). We extend this research in vehicle control to robot teleoperation, investigating how to prime different perceptions of the robot, and if the priming affects teleoperation performance.

Social human-robot interaction has explored the use of priming, or a variant called framing (Westlund et al., 2016; Sanoubari et al., 2018; Liu, 2020; Wallkötter et al., 2020) in social interactions between people and social robots. More generally, it has been argued for some time that user expectations and perceptions are crucial for shaping their expectations and willingness to interact with autonomous social robots (Lindgaard et al., 2006; Mitra and Golder, 2006; Young et al., 2009; Edwards et al., 2019; Reich-Stiebert et al., 2019; Vattheuer et al., 2020). In the priming direction, some work has shown how subtle shifts in language used to describe a robots can influence how personal (Coeckelbergh, 2011) or human-like (Anna et al., 2012) people view or treat the robots (Sam and Tom, 2017). Others have shown how priming can be used to encourage people to believe an autonomous robot is actually teleoperated by a human (Tanaka et al., 2016), altering how they engage with the robot. People will even subconsciously imitate robot speech patterns when interacting with a robot (Brandstetter et al., 2017), an effect called lexical entrainment that shares similarities to priming. In our work, we apply this priming approach specifically to teleoperation of robots.

For teleoperation, a body of research not explicitly done under the umbrella of priming uses stimuli to evoke feelings and influence behavior. For example, altering a robot’s acceleration and speed curves (to *feel* more or less heavy, for example) can impact operator mental workload and performance (Rea et al., 2017a; Praveena et al., 2020), improve feelings of safety (Basu et al., 2017), or change operator emotions (Rea and Young, 2019a; Rea et al., 2020). Subtle haptic feedback mechanisms (perhaps not consciously noticed by the operator) that reflect the remote robot’s environment can influence operator performance (Hacinecipoglu et al., 2013). Overall, the works in this section highlight the broad potential and range of possibilities for influencing and shaping operator psychology and ultimately their behavior. We continue this direction by specifically investigating how to shape teleoperator perceptions about their robot’s physical abilities, ultimately to support effective operation.

## 4 Novel teleoperator priming techniques

**FIGURE 2:**
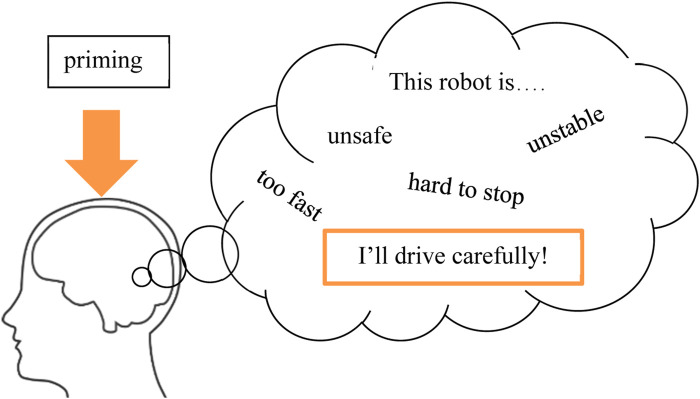
Our experiments test three different priming methods (including no priming) and observe their effects on an operator’s driving behavior and perception of their robot.

Our high-level goal is to generally investigate the feasibility of using priming techniques to shape operator perceptions about a robot and its capabilities, and how they may operate the robot. For our early exploration, we focus on priming stimuli that suggest how *safe*, or *unsafe*, a robot may be, thus potentially instilling beliefs into an operator and changing how they operate the robot (Figure 2), building on prior research suggesting how perceptions of safety may impact driving behavior (Groeger and Rothengatter, 1998; Groeger, 2002).

To achieve this, our priming strategy was to convey properties of the robot’s driving ability relating to safety, such as how powerful the motor is, how easy it is to steer, and how durable the robot is (e.g., when colliding with the environment). For each priming method, we developed three interface instances along a continuum, with one method suggesting *unsafe* robot characteristics to a user, one suggesting *safe* characteristics, and one somewhere in the middle. We emphasize that in *all cases, no actual properties of the robot or its response to commands (speed, ability, etc.) changed*–in each case the same command (joystick pitch and yaw) created the same response in the robot. Thus, we can study the impact of the priming method independent of robot performance.

It is not clear which approach–safer *versus* less-safe robot–would result in better driving. One could imagine operators would drive better when primed that the robot was *unsafe*, to compensate for the expected poor performance, and drive worse with the *safe* robot as they feel less pressure to be careful. Inversely, perhaps the impression of *safe* or *unsafe* would encourage them to act likewise, where simply thinking about safety (or lack of) may make the person drive safer (or less so) by either relying on the perceived safety and not taking precautions, or using perceived lack of safety of the robot as an excuse for their own performance. Our hypotheses on the impact of priming is non-directional: we do not hypothesize what the direction of the impact will be.

We explored two different approaches: tangible priming (a continuous, physical indicator of robot ability), and descriptive priming (a verbally and visually explained, cognitive indicator of robot ability).

### 4.1 Tangible priming

Our strategy for tangible priming was to convey robot ability through the tangible response of the control method. Our hypothesis was that a control method that takes more effort to use would convey a sense of a heavier, slower robot, which is safer to drive. Conversely, a control method that requires little effort to use would convey a lighter, faster robot, that may be unsafe and easier to crash and break.

The tangible method fits the model of priming where a constant, ongoing stimulus is provided–see Related Work, and examples such as (Henk Aarts and Dijksterhuis, 2003; Ackerman et al., 2010). Instead of a single, up front priming stimulus, our tangible priming method continuously reminds the operator of their experience and prior knowledge which may then continuously evoke a priming effect (Bargh et al., 1996).

Specifically, we used different spring stiffnesses of a joystick used to drive the robot to impart this tangible feel. We used three static settings for joystick stiffness, one per condition: high stiffness (to convey a heavier, slower, and thus *safe* robot), low stiffness (to convey a lighter, faster, and thus *unsafe* robot), and a mid-point in between. Note the stiffness was fixed per condition (static) and did not change during operation. In all cases, the joystick stiffness provides a constant reminder of the robot’s ability.

A key element of priming is how the technique is introduced to operators. We simply told people that “each robot will interact with the joystick differently, based on the robot’s physical design.” Our goal was to avoid telling people what our intended impact was (i.e., safe vs. unsafe robot), but to let them know that joystick changes were intentional and did relate to the robot capability, letting participants decide what is safe.

**FIGURE 3:**
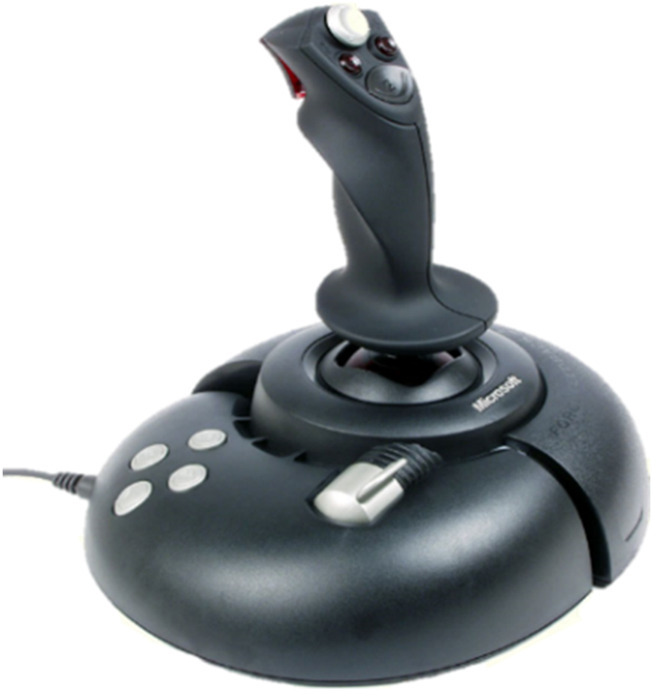
The joystick we used for tangible priming–Microsoft Sidewinder Force Feedback 2 USB joystick. It can be dynamically programmed to have different stiffness settings.

We implemented this technique using a force-feedback joystick (Figure 3), which has a programmable stiffness setting. We used 100% of device maximum spring strength and friction for the safe condition, 10% for the unsafe condition (0% would not provide enough stiffness to naturally return the joystick to a neutral centre position), and 50% for the middle case. The strongest setting (*safe*) took noticeably more force to operate than a regular joystick but, was not onerous to operate and we did not anticipate fatigue to be an issue. The weakest setting (*unsafe*) was strong enough to automatically return to a cantered position after being pushed but put very little force onto the user. The robot response to a given joystick input (pitch and yaw values) did not change: a given joystick position would result in identical behavior regardless of stiffness settings. We remind readers that while operators were told they would be testing different robots, secretly the robot and its capabilities were never changed–just the priming stimulus.

### 4.2 Descriptive priming

**FIGURE 4:**
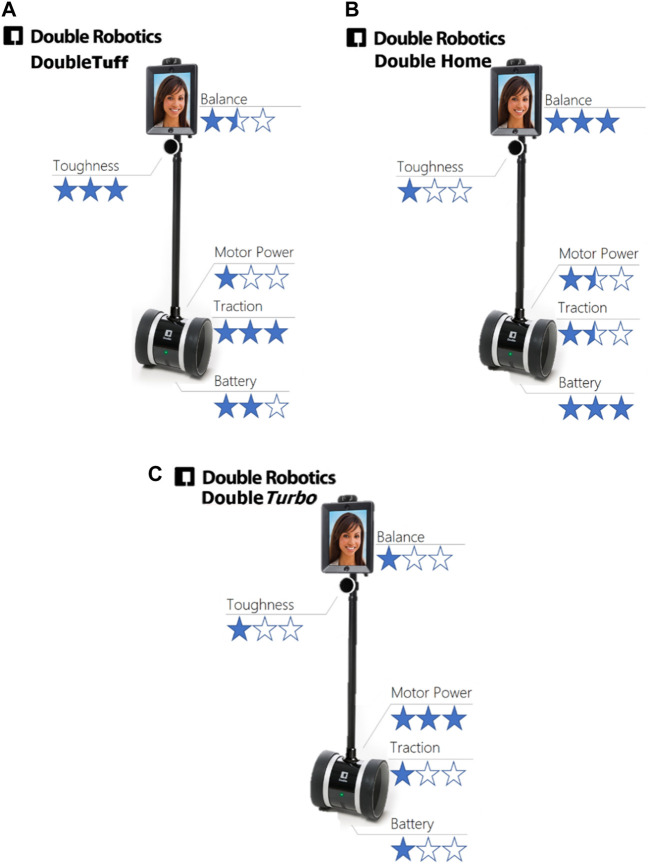
These descriptive priming sheets were designed to shape operator expectations before operating the robot. Note these were not in the interface itself, but simply presented as part of the study protocol. **(A)** the safe condition **(B)** the middle condition **(C)** the unsafe condition.

For this method we investigated if priming by altering how we describe a robot to an operator would impact perceptions of robot ability after operating the robot. We employed both verbal description and visual aids (Figure 4) that explicitly define specific robot performance characteristics, overall creating three robot instances falling on a continuum from safe to unsafe. In this case, no tangible priming was employed.

We achieved the impression of safety by describing four robot characteristics, selected as attributes that we expect non-expert operators to easily understand and relate to operation safety. These were robot “balance,” “toughness,” “motor power,” and “traction.” We further added to our description a non-safety item (“battery”) to help avoid participants guessing the study purpose.

We presented this information on paper (Figure 4), along with a scripted explanation for introducing each robot and variable that emphasized the safety and risks of each, but without explicitly telling operators our purpose. The labels and the descriptions we use are described in Table 1.

**TABLE 1:** A list of the descriptively primed robot properties and how we explained them to participants.

Name	Description: “A robot’s ability to … ”
Balance	Stay upright easily, regardless of surface, obstacles, or operation
Toughness	Not be damaged from collisions
Motor Power	Accelerate quickly to a high top speed
Traction	Turn quickly and safety
Battery	Continue operating for long periods of time

We told operators that these measures are derived from a number of components in the robot, as rated by the manufacturer, and we further gave the robot names to suggest their safety level (Figure 4). People kept the relevant specification sheet in front of them during operation. Again, they secretly always drove the same robot, and were only primed to believe it was different.

## 5 Initial two studies: tangible and descriptive priming

We conducted two initial studies to investigate the impact of each of our priming methods on teleoperation (previously summarized in Rea and Young 2018). We do not analyse this as a single study (with priming method as a between-subjects variable) given that we first completed the tangible study, with the descriptive condition following up at a later time: participants were not time-wise balanced between conditions, and we further introduced minor changes (explained below). As such, we present and analyze our results as a series of two separate studies.

We conducted within-participants studies where a single participant completed a task with all three robot conditions (safe, unsafe, in-between). A within-participants design enabled participants to directly compare and contrast the robots between priming conditions, and further provided more statistical power by factoring out individual differences in driving ability, susceptibility to priming, *etc.*

A key element of our study design was to give people a representative experience operating the robots; particularly for the descriptive cases, participants need enough experience so that they do not simply report back on what they were told. Ostensibly, after driving each robot for a period of time we could reasonably expect participants to notice that the robots were the same (or at least very similar), despite the priming stimulus.

### 5.1 Task

**FIGURE 5:**
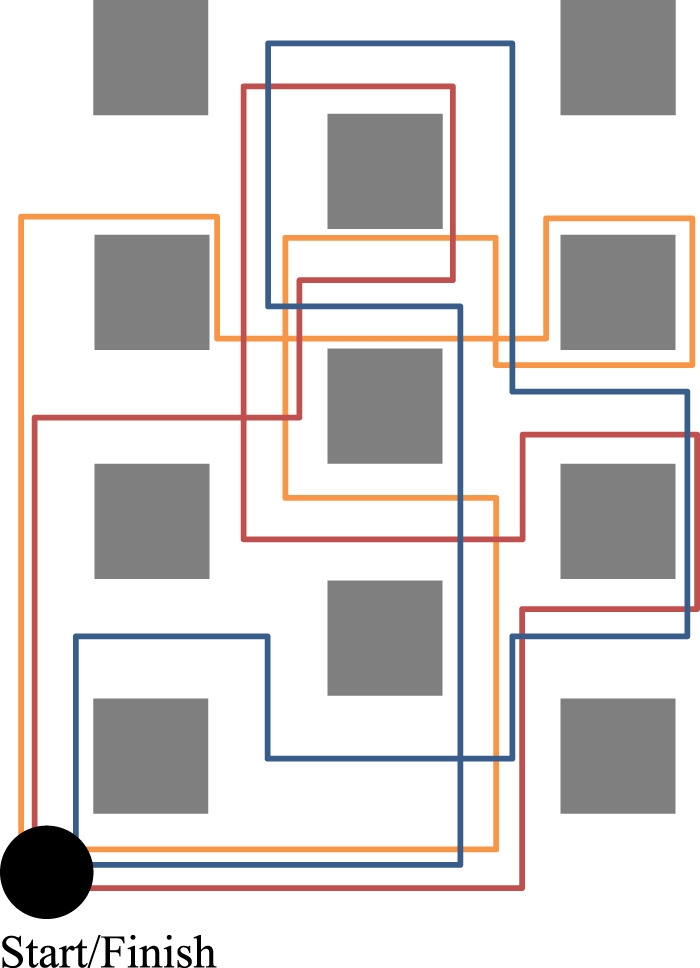
The room and obstacle layout used in the study design, with the three paths through it. Experimental conditions were balanced across courses to mitigate effects due to differences in the course design.

**FIGURE 6:**
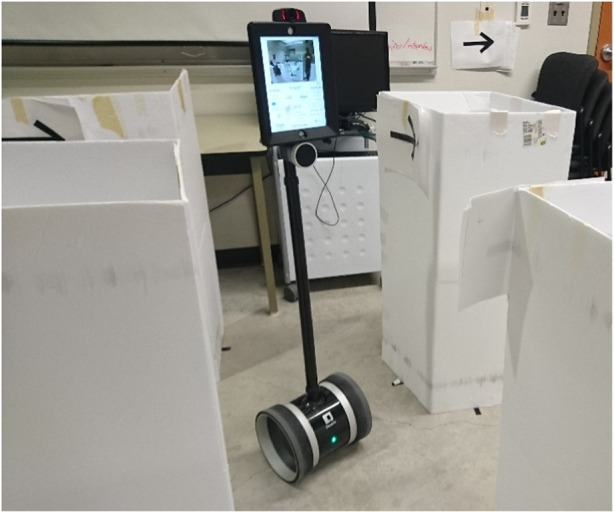
A robot is driven through an obstacle course. We primed operators to believe that they were driving robots with different capabilities and potential risks. However, the robot secretly never changed. We examined how priming changes teleoperation behavior and perception of the robots.

We tasked participants with navigating a telepresence robot through an obstacle course (Figure 6). They were instructed to drive and complete the task as quickly as they felt comfortable, while trying to avoid colliding with obstacles, walls, *etc.* As each participant completed three conditions (safe, unsafe, in-between), we created three paths through the course with all having the same difficulty: same number of turns and distance (Figure 5). Each path took approximately 2–5 min per lap, depending on driving speed, the number of collisions, and overall participant skill. For each condition, participants were asked to first complete a training lap, followed by two laps for the study. Condition order was counter-balanced across participants and courses. The obstacles are equally distant from each other and were only slightly wider than the robot itself (Figure 6). The experiment setting was designed to be difficult to perform at 100% safety (no collisions).

### 5.2 Instruments

Participants operated a Double 2 robot (Double Robotics) with a 150° field-of-view camera. The robot’s camera feed (640 × 480 pixels) was viewed full-screen (with black bars on the wide-screen sides) on 24-inch monitor, in a separate space from the robot, and participants were seated at roughly the same position with respect to the monitor. The system maintained at least 15 frames per second, but was as fast as 30 frames per second, depending on network health.

While driving the robot, participants wore headphones that relayed sound from a microphone mounted on the robot in the remote space. Participants used a Microsoft Sidewinder USB Force Feedback 2 joystick for both studies, with stiffness set for tangible priming as explained above, and fixed at 50% for the descriptive study.

Participants completed questionnaires (detailed in the next subsection) on a separate monitor using Google Forms.

### 5.3 Measurements

Our performance measurements were selected as simple teleoperation measures used in prior work (Jessie et al., 2007; Rea et al., 2017a)–completion time, collisions, and perceived workload. We additionally measured teleoperator perception of the robot and its physical capabilities.

Pre-experiment, we gathered demographics information to better understand the variance in our sample. We collected information including age, gender, frequency of playing video games, frequency of driving, and self-reported driving skill.

For each condition, a researcher in the room with the remote robot measured completion time and collisions. Perceived workload was measured post-condition with the NASA Task Load Index or TLX (Hart and Staveland, 1988) self-report questionnaire. To get a sense of a participant’s perceptions of a robot’s capabilities (and the effects of our priming) we also administered 5-point Likert-like scale items inquiring about a participant’s opinions on the robot’s speed, weight, steering, durability, power, safety, and responsiveness. Participants then completed free-form written questions inquiring about their experience. These questions were optional, and asked participants for any positive, negative, or other feedback they wished to provide us about the robot and teleoperation experience.

### 5.4 Procedure

The same procedure was followed for both studies, with differences highlighted in the corresponding sections below. Participants were first given a briefing of the experiment and signed an informed consent form. Participants were told that they will test 3 new prototype telepresence robots in order to help us evaluate the safety and drivability of each robot for new users but were not told specifically that the robots being designed for different safety levels. This was a deception–in reality the participants *used the same robot in each primed condition*. We described the robots as being similar in size and shape, but with different internal components that may change how they perform.

We explained the overall procedure of the experiment and introduced the joystick and obstacle course. Further, before starting, we explained either the connection between the robot and joystick (for tangible priming), or a high-level overview of the robot data sheets (for the descriptive priming), as explained in our priming method overview. The participants were seated in a room separate from the robot and obstacle course.

Following the introduction, each participant completed the task three times, once per priming condition (safe, middle, unsafe), with the order of the priming conditions and the path through the course (Figure 5) counterbalanced. Before starting each of the three conditions, participants were first asked to complete a training lap, before the main two laps of their task. This training allowed participants to become familiar with the new obstacle course (and reduce confusion from the new course) and gave them additional practice with the “new” robot. This practice added to our priming story–participants believed they were operating a new robot, and we told them that the training was for them to get used to the differences between each robot model.

After each of the three conditions, we administered the post-condition questionnaires described earlier (NASA TLX, perception of robot abilities). To transition between conditions, we disconnected the robot from the control interface to give the illusion of switching to a new robot, although the same robot was reconnected upon starting the new condition. As the participant was in a space separate from the robot, we were able to maintain this illusion.

Post-test, participants were debriefed about the priming purpose and the deception (that it was a single robot only). The experiment was then re-explained in the context of the deception and how the deception helps achieve the research goal. The participants were encouraged to engage with a discussion with the researcher about the experiment. Our university’s research ethics board approved all studies.

### 5.5 Study: tangible priming

For the tangible priming study, we recruited 25 participants; however, one did not complete the experiment due to technical issues. Two other participants were identified as outliers: we observed them not attempting to avoid obstacles (e.g., laughing and pushing obstacles around seemingly on purpose), and this was reinforced from their data (>1.5 Inter-quartile range). This resulted in 22 participants (mean age of 24, standard deviation of 6.3 years; 12 female).

#### 5.5.1 Results: tangible priming

To investigate whether the tangible priming worked, we conducted Friedman’s ANOVA tests on our Likert-like scale perception data. We found statistically significant results for perceived speed, perceived steering ability, perceived durability, and perceived safety (Table 2). The perceived safety results matched our expectations that the stiffer joystick would be seen as more safe, acting as a manipulation check. Other tests on perceived experience were not significant. We found no effect of variables from the demographics questionnaire (video game, driving experience) on any of our measures.

**TABLE 2:** Mean ranks and chi-square values for perceptual effects for tangible priming.

	unsafe	middle	safe	χ2 (2)	p	perceptual effect as spring stiffness increased
Speed	2	2.4	1.7	7.0	.03	(mixed) lower speed
Steering	1.6	2.2	2.2	6.6	.04	Improved steering
Durability	1.7	2	2.3	6.9	.03	Improved durability
Safety	1.7	2	2.3	8.0	.02	Improved safety

Higher ranks for steering, durability, and safety are considered “better” and higher ranks for speed are considered “faster”. For example, safe is considered the slowest with better steering and durability than unsafe. All listed values are *p* < .05. Omitted variables are n.s. (mixed) indicates that the middle condition was not the midpoint of the 3 conditions.

Both completion time and number of collisions were right skewed (non-normal, Shapiro-Wilk test, *p* < .05), and were corrected using a square root transform.

**FIGURE 7:**
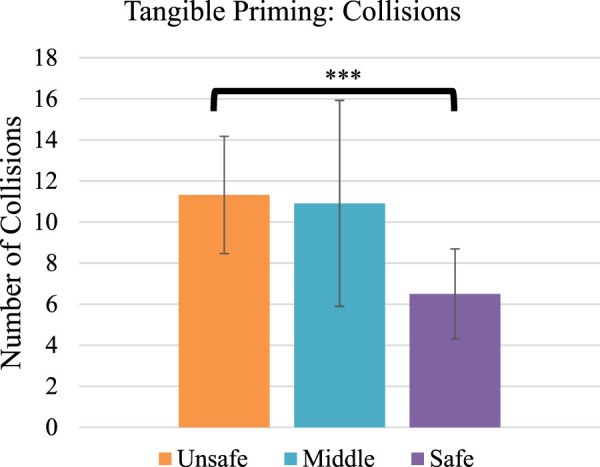
Average collisions per condition. ***p < .001. Error bars show 95% confidence interval.

To investigate performance, we performed repeated-measures ANOVAs on completion time, collisions, and perceived workload. We found a statistically significant, medium effect of tangible priming condition on collisions (F_2,42_ = 5.2, *p* = .01, η^2^ = .20, Figure 7). Post-hoc tests (Bonferroni familywise correction) found the safe condition to have on average 4.8 fewer collisions (42% fewer) than the unsafe condition [*p* = .001, 95% confidence interval of the mean difference (1.8 collisions, 7.8 collisions)].

**FIGURE 8:**
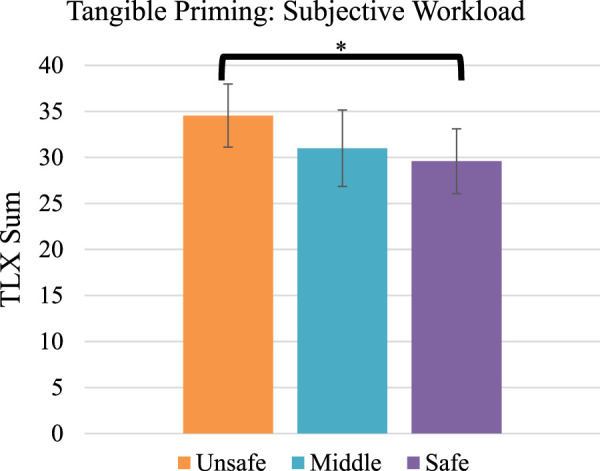
Average TLX sum score per condition. *p < .05. Error bars show 95% confidence interval.

We further found a statistically significant medium effect of tangible priming on perceived workload (NASA TLX sum, F_2,42_ = 3.6, *p* < .04, η^2^ = .14, Figure 8). Post-hoc tests (Bonferroni familywise correction) found the non-safe condition to have on average 5.0 points higher (14% higher) perceived workload than the safe condition [*p* < .04, 95% confidence interval of the mean difference (.22 TLX points, 9.7 points)]. We did not detect a difference in completion time (*p* > .05).

#### 5.5.2 Discussion of tangible priming

Our results indicate that our tangible priming conditions caused participants to perceive the robot and teleoperation experience differently: we found differences in perceived safety, durability, steering ability, and speed. Further, the difference in perceived safety confirmed the intended manipulation of our priming design was successful. Given that the robot reacted and responded identically in all conditions, and participants spent time controlling the robot, if the priming was not effective it would be reasonable to expect participants to rate the robots based on how it actually performed, and perhaps notice that the robots were the same or similar. However, the fact that participants rated the robots differently despite this is a clear indication that the tangible priming method worked to shape participant perception of the robot and teleoperation experience.

We further found a significant difference in collisions, with the non-safe condition having a 42% reduction (average 11.4 in the unsafe, and 6.6 in the safe), and participants reporting lower task load with the safe condition (average 5.0 TLX points, 14%, lower than the unsafe condition). We cannot speak to the exact mechanism by which our tangible priming method may have caused this improvement in driving: perhaps the priming encouraged people to drive more slowly, take fewer risks, or take wider turns around obstacles. Further study is needed to understand the specific mechanisms and how they produce the effect.

Looking at our performance and perception results together, we see that people drove the safe condition in a safer manner and perceived it as safer than the other conditions. While some related work suggests people may drive a safer vehicle more recklessly (Jonah et al., 2001) we reemphasize that, in our specific implementation, we had plausible explanations for either an increase or decrease in safety and thus did not hypothesize a specific direction of effect (see our priming technique overview).

Regardless, our priming method was a success, considering the changes in perception (e.g., decreased speed or improved steering capabilities in the safe condition) when participants drove an identical robot each time. We conclude that the physical properties of an input method can be used to prime users and change their perceptions of the robot and may also impact their performance.

We note, however, a potential confound in the study: the *usability* of the different stiffness settings may explain the performance difference. That is, perhaps the stiffer joystick was simply easier to use than the looser setting, explaining the reduced collisions, and thus the improved perception of safety. Before addressing other future work, such as the mechanisms of priming itself, we re-visit this issue in a follow-up study presented later in this paper (Section 6).

### 5.6 Study: descriptive priming

We recruited 24 participants (none participated in the Tangible Priming study); three were removed as outliers as they did not attempt to avoid obstacles (e.g. driving full speed and not stopping for any obstacle) or did not appear to understand the instructions (e.g., frequently took wrong turns in the obstacle course). This was reinforced as outliers in the data (>1.5 inter-quartile range). This resulted in 21 participants (mean age 24, SD 6.3 years; 12 female).

The priming specification sheets (Figure 4) were explained in detail to participants at the introduction of the study, and the sheet associated with each condition was left with the participant during the task. Participants were given time to review the specification sheet (the priming) before each condition, and the sheets were removed during the post-condition questionnaire.

In the tangible priming study, we noticed a subjective improvement to participants’ performances as the study went on, due to, we presume, becoming more skilled at operating the robot. While this improvement was mitigated somewhat in our results due to counterbalancing and initial training lap, to further reduce potential learning effects we added an additional up-front training step after the initial explanation, and before the first condition: participants practiced using an additional, similar path through the obstacle course for two laps. Participants were told they were piloting the current commercially available robot model (compared to the “prototypes” that followed).

Additional self-report measurements were added post-experiment to reflect the details of our priming. Participants rated the robots on the criteria we used in the priming specification sheets (Figure 3), asking what their impression was of the robot’s motor power, traction, balance, toughness, and battery life was. Participants were specifically asked to report based on their teleoperation experience, not on their memory of the specification sheets. This final questionnaire was completed on paper. We remind readers that only the information provided in the information sheet differed between conditions (not the robot), though participants were led to believe they were testing different robots.

#### 5.6.1 Results: descriptive priming

To investigate whether the priming worked, we conducted Friedman’s ANOVA tests on our post-condition Likert-like scale data. We found statistically significant results for perceived speed, perceived steering ability, perceived durability, and perceived safety (see Table 3). Other tests on perceived teleoperation experience were non-significant. Friedman’s ANOVA tests on the post-experiment specification sheets found statistically significant results for balance and motor power, with trends for toughness and traction. These results are also included in Table 3.

**TABLE 3:** Mean ranks and chi-square values for perceptual effects for descriptive priming. Omitted tests are n.s.

	unsafe	middle	safe	χ2 (2)	p	perceptual effects as increased safety was primed
Speed	2.5	1.5	1.9	8.6	.01	(mixed) lower speed
Weight	1.6	2.1	2.3	6.5	.04	higher weight
Power	2.4	1.7	1.9	7.5	.02	(mixed) less power
Safety	1.6	2.3	2.1	8.3	.012	(mixed) more safe
Balance	1.6	2.5	1.9	12.7	<.01	(mixed) more balanced
Motor power	2.4	1.6	2.0	7.4	.03	(mixed) less motor power)
Toughness*	1.7	2.0	2.3	4.6	.10	tougher
Traction*	1.6	2.1	2.2	4.9	.09	Improved traction

Higher ranks for power, balance, toughness, and safety are considered “better” and higher ranks for speed are considered “faster”, and higher ranks for weight are considered “heavier.” (mixed) indicates that the middle condition was not the midpoint of the 3 conditions.

*marked measures are statistical trends of interest, but not significant.

**FIGURE 9:**
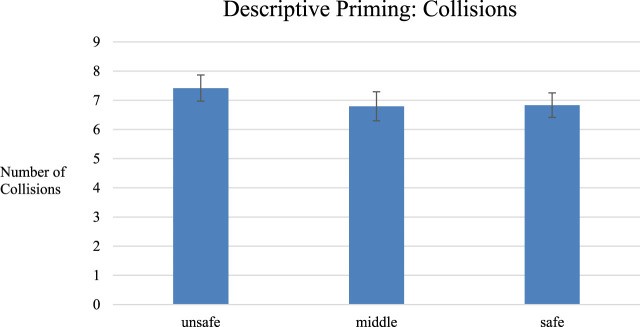
The collision results from Descriptive Priming. Results are n. s. Error bars show 95% CI.

With repeated measures ANOVAs we found no significant results on completion time (F_2,38_ = .2, *p* = .83, η^2^ = .01, means for unsafe = 165s, middle = 176s, safe = 171s), collisions (F_2, 38_ = .2, *p* = .68, η^2^ = .01means for unsafe = 6.0 collisions, middle = 5.4 collisions, safe = 5.8 collisions, see Figure 9), and perceived workload (F_2,38_ = .7, *p* = .48, η^2^ = .04, means for unsafe = 29.4 points, middle = 29.4 points, safe = 27.7 points).

#### 5.6.2 Qualitative results

Given the lack of impact of description priming on teleoperator performance, we performed *post hoc* open-coding qualitative analysis on participant short-form responses to learn more about operator driving experience. Coding was done with a single coder with thematic analysis; the purpose of this analysis was not to make definitive conclusions about why participants acted in a given way, but to better understand how and why participant’s may have rated the robot’s perceived abilities differently, to inform follow-up work.

We found that 20 participants (83%) made explicit comparisons between the robots’ capabilities and their teleoperation experiences with them:

I love the response time and the power of the (unsafe condition). It is quicker than the (safe condition) and I felt like the wind.–p9
I felt more in control with (the safe condition)–p19
Aside from durability, everything else about (the middle condition) felt more stable–p14.

These comments covered a range of aspects of teleoperation, which we found to reflect consistent opinions of a robot’s perceived abilities across conditions. Further, these comments aligned well with the primed robot characteristics.

All eight participants who mentioned speed wrote that the unsafe condition was faster than other robots:

It is quicker (unsafe condition) than the previous robot and I felt like the wind–p33.
It was hard to keep the balance on this robot (unsafe condition) as it was light and had more speed.–p16.

Speed was less commonly mentioned in the other conditions (three times total), which were characterized as slower:

(The middle condition) did not accelerate as fast as the other robots–p2.

Control was another common theme, where six people reported the safe condition as having better control:

I liked how in control I felt of the steering and acceleration. There were no surprises.–p11.

There was one comment with a negative opinion of the control of the safe condition. In contrast, three people mentioned that the middle condition had better control than the unsafe condition, and two mentioned that the unsafe condition had worse control overall.

Finally, “responsiveness” was another common theme. The unsafe robot was most commonly discussed, with seven participants saying that it was more responsive, for example:

It responds quickly, and seemed to navigate at relatively high speed.–p13.

The four participants who mentioned responsiveness with the middle safety robot all had comments similar to:

The robot felt more flimsy and unresponsive–p11.

Only two participants mentioned the responsiveness of the safe condition. One participant mentioned it was “more responsive”–p22, while the other disagreed:

The robot is slower, does not have a faster response rate, motor power is definitely weak. My head is hurting trying to operate this robot–p9.

#### 5.6.3 Discussion of descriptive priming

In this experiment, we investigated the impact of priming teleoperation operators using a visual and verbal description of the robot. Our results suggest that descriptive priming (using paper and speech only) successfully changed participant perception of the robot, and their experience teleoperating it, even after operating it for some time. We successfully altered participant perception of robot speed, weight, power, and overall safety. We note that the middle safety condition often performed the highest or lowest for a perceptual measure–the reasons for this are unclear and require future work. However, our post-test questionnaire results indicated that our non-safe condition was successfully primed to be seen as riskier than our safe condition in terms of balance and motor power, with trends pointing to potential priming in toughness and traction. These results emerged despite participants driving the exact same robot in each condition.

Our qualitative results further supported this and highlighted the effectiveness of our priming. More than simply memorizing the details provided to them, the conviction and tone in the written feedback suggests that the participants believed that the differences were real, despite having operated the exact same robot through a task repeatedly.

We did not find any performance change in terms of completion time, collisions, or perceived workload. It is possible that there is still a small effect that went undiscovered due to our small sample size of 21. If there is indeed no effect on performance, it will be important to further investigate how this disparity between perceptions and performance can happen, and what it means for long-term use.

Importantly, our results suggest that we can improve user perception of the safety or physical capabilities of the robot without sacrificing performance or changing functional aspects of the design. In fact, we highlight that descriptive priming had these effects with only an information sheet being distributed to participants, easily implemented with any robot product. As such information is often already printed in user guides, this study suggests that extensive care should be given to such materials as they may significantly impact perception and expectations of the robot even after extended use.

### 5.7 Reflection on tangible and descriptive priming

Both priming methods were effective at changing the user’s perception of the robot, while the actual experience of driving the (secretly identical) robots did not seem to counteract the priming. That is, even after driving the identical robots themselves for multiple trials and training, for upwards of 30 min, participants rated the robot capabilities differently, but similarly to how we primed them. Both methods primed changes in perception of a robot’s speed and safety, but there were differences in perception of the robot between the two methods: tangible priming changed perceived steering and durability, and descriptive priming changed weight and power (Table 6). While this makes sense for the descriptive priming case–it matches our priming focus–for the tangible case the connection to durability is less clear. Further, we observed a difference in actual driving performance for tangible priming, with the stiffer joystick (safe priming) resulting in, on average, 4.8 fewer collisions than the looser joystick (unsafe priming). This highlights the need to consider and the technique used to prime, and how choices may inherently work well for some perception and behavior outcomes and not others.

**TABLE 6:** The perceptual rankings of the tangible and descriptive priming studies have been reproduced here for comparison.

	Tangible Priming			Descriptive Priming		
	Loose	Middle	Stiff	Non-safe	Middle	Safe
Speed	2	2.4	1.7	2.5	1.5	1.9
Steering	1.6	2.2	2.2	1.6	1.8	1.8
Durability	1.7	2	2.3	1.5	2.6	1.8
Safety	1.7	2	2.3	1.6	2.3	2.1

Note that for Desciptive Priming, the durability and steering measures were not found to be significant, though all other ratings here are.

It is worth considering further why only the tangible case impacted driving performance. First, we note that the tangible condition also resulted in a difference in operator perceived workload, with the safe condition resulting in a 14% reduction (in TLX score) compared with the unsafe condition; no difference was found on workload with descriptive priming. Perhaps one reason is that the tangible priming is directly linked to control (being the joystick) while the description is more abstract. Or, perhaps this is due to the tangible priming being a more salient constant reminder of the priming in comparison to the descriptive paper which just sat beside the joystick, while the participant was busy with the task. These questions about the mechanisms via which priming created its effects require further study.

Another possibility is that the impact on driving performance may not have been due to the priming. Perhaps *the joystick stiffness itself* has a usability impact, where one joystick (in this case, the stiffer one) is simply easier to control than the other (the less stiff one). If that is the case, then it is the joystick usability–and not our priming method–which may be responsible for the driving performance and workload result. We conduct a follow-up study (detailed in the next section) to explore this possibility.

Overall, we feel that these two studies were a success. We were able to leverage priming to consistently change operator perceptions of the robot, perceptions which persisted even after using the robot for upwards of 30 min. While the impact on actual driving performance was mixed, we note that shaping perceptions itself is an important element of interface design (Young et al., 2009), as it can shape expectations, user workload or stress, and affect technology adoption on the long term.

The above results were previously published (Rea and Young, 2018; Rea and Young, 2019b), but were included for discussion with the follow-up study below.

## 6 Follow-up study: joystick stiffness–priming or usability?

We conducted a follow-up study specifically to test the usability component of our tangible priming method, which used joystick stiffness to represent robot capability. That is, we inquired whether joystick stiffness impacts the usability of the joystick as a robot control method in a way that could explain our tangible priming results. We investigated if a stiffer joystick is simply easier to control than a looser joystick. Such a result would require us to re-analyze our results from our tangible priming study above, as it would suggest that the usability of joystick stiffness–not the priming it induces–may explain the improved driving performance.

Our approach was to replicate our tangible priming study while removing the priming (and thus deception) by clearly explaining the joystick stiffness manipulation to participants and telling them the robot was always the same. That is, instead of us leading participants to believe that the joystick stiffness reflects robot ability and weight, we instead simply tell them that the robot does not change, only the joystick setting. Analyzing this alongside the results from the tangible priming study enables us to separate the effects of the joystick usability from priming effects. On the one hand, if we still find the same effects without the priming, then we can conclude that it was the usability–and not the priming–that explains our results. On the other hand, if we do not find an effect of the joystick stiffness on teleoperation performance, then this lends support to our conclusion that priming is the driver of our earlier results.

### 6.1 Procedure

We use the same procedure as explained for the tangible priming experiment. The primary difference was we did not tell participants that the joystick stiffness represented the robot’s capabilities (priming). Instead, we explicitly explained the study conditions to the participants–that they are driving the same robot repeatedly, and that the only thing we change is the joystick stiffness. We explicitly said that, although the joystick stiffness changes, the robot’s response to the joystick does not change: a given joystick movement or position will result in the exact same robot reaction, regardless of stiffness setting.

Participants first completed the same pre-test demographics questionnaire, before being introduced to the system. All conditions were explained (as above), and participants completed three conditions, with the same three joystick stiffness settings used in the tangible priming study (with the same counter balancing). To maintain consistency with the original tangible priming study, the extra training session before the experiment (added in the descriptive priming experiment) was not included.

Each condition consisted of a training lap, followed by two laps that were recorded. During the condition we recorded completion time and collisions, and after each condition we administered the perception questionnaires from our tangible priming study. We re-emphasized to participants before each condition that we were only changing the joystick stiffness. Post-experiment, we elicited general qualitative feedback (as in previous studies), and debriefed and discussed the experiment with participants.

### 6.2 Results

We recruited 18 participants (mean age 24, SD 9.4 years; 12 female)–none participated in the prior descriptive tangible or descriptive priming studies.

To investigate teleoperation performance, we performed repeated measures ANOVAs on completion time, collisions, and perceived workload; we found no significant results for any of the three variables (summarized in Table 4).

**TABLE 4:** ANOVA results for our three main performance measures with no priming.

	F-ratio	p	η^2^	Not safe	Middle	Safe
Completion time (s)	F_2,34_ = 0.6	.56	.03	208	213	219
Number of collisions	F_1.4,23.8_ = 2.9	.07	.14	6.3	7.2	8.2
Workload (TLX score)	F_2,34_ = 2.2	.12	.11	32.1	28.3	30.0

To investigate if there were any priming effects on operator perception of the robot, we conducted Friedman’s ANOVA tests on our post-condition Likert-like scale data. We found statistically significant results for perceived weight, perceived steering ability, perceived durability, and perceived safety (see Table 5). Other tests on perceived teleoperation experience, including perceived workload (NASA TLX), were non-significant (see Table 4).

**TABLE 5:** Mean ranks and chi-square values for perceptual effects for no priming.

	unsafe	middle	safe	χ^2^ (2)	perceptual effects as spring stiffness increased
Speed	2.1	2.2	1.7	3.6	(mixed) decreased speed
Weight*	1.4	2	2.7	17.4	increased weight
Steering*	1.5	2.4	2.1	11	(mixed) improved steering
Durability*	1.6	2.2	2.2	7.6	improved durability
Safety*	1.6	2.4	2.0	9.8	(mixed) improved safety
Responsive	2.1	2.2	1.8	2.4	(mixed) decreased responsiveness

*p < .05. Higher ranks for power, balance, toughness, and safety are considered “better” and higher ranks for speed are considered “faster”, and higher ranks for weight are considered “heavier.” (mixed) indicates that the middle condition was not the midpoint of the 3 conditions.

### 6.3 Discussion–priming or usability?

We found no statistically significant impact of joystick stiffness on any measure of driving performance in this no-priming study, which contrasts the findings in our prior tangible priming study (Section 4.1).

First, we considered the possibility that our study was under-powered and simply required more participants. However, the statistics provide no indication of this (e.g., for completion time we have an F-ratio of less than one, with a very small η^2^). While collisions could be considered a trend with a medium effect (*p* = .07**,** η^2^ = .14), the effect was *opposite* of the prior study (stiffer joystick had more collisions), and the actual differences observed were much smaller (on average 1.9 collisions, Figure 9, *versus* 4.8, Figure 7), suggesting that the three joystick stiffness levels were either similar in this un-primed case, or the stiffer joystick made participants drive *less safely*, as opposed to the primed experiment where they drove more safely.

The lack of a workload difference in our no priming study also supports our original conclusion that the difference in workload was due to our priming method. However, we saw an F-ratio of 2.2 with a medium effect size (η^2^ = .11) and a roughly similar trend of lower workload as stiffness increased. This suggests a small effect may be seen with more participants, and that usability may play at least a small part in our tangible priming workload change. However, this requires future study, and we emphasize that this result is inconclusive for workload.

In the tangible priming case, operators reported feeling a difference in the driving feel (change in workload) and did drive differently (a significant change in number of collisions). In the no-priming case, operators did not feel the workload was different, and we did not detect a difference in performance. If change in joystick stiffness really was a major usability factor and usability created the reduction in collisions we originally observed, we would expect it to be reflected in both studies. Our observations did not see similar changes in our no priming study. While it is possible we simply did not detect a smaller effect, our data leads us to conclude that it was likely our priming method primarily (tangible priming), and not the usability of the device, that resulted in at least some of the improved driving performance. However, we still saw similar changes in perceptual measures in the no-priming case (increased safety-related perceptions with a stiffer joystick), leading us to question if we really eliminated all priming effects, despite telling participants exactly what was happening. We combine these potentially confusing results with the prior studies in our discussion.

## 7 Overview: cross-study discussion on teleoperation priming

In this work, we explored two priming methods, and conducted a follow-up study to further investigate and isolate one of our priming variables. Specifically, we investigated the impact of our priming *versus* the actual usability of our tangible priming interface. Through this 3-study exploration, we established that our priming techniques can indeed shape operator perceptions, and in some cases, the priming can improve operator driving performance. However, our results further highlight some caveats to these conclusions.

First, despite multi-study impact on perceptions of the robots, operators’ driving performance was only impacted in a single case (tangible priming)–we anticipated broader impact. Second, we note that operator perceptions were shaped in *all* cases, even when we attempted to remove the explicit priming element of our study (and thus would expect to see no impact on perceptions); thus, it may be problematic to attribute our results (on how operators perceived the robots) solely to our priming methods. We need to take a deeper look at our results to consider why the impact of priming was not more consistent and predictable throughout our studies.

### 7.1 Driving performance only improved for tangible priming

Despite our initial motivations to shape operator perceptions and behavior broadly, in our three experiments we only found operator performance increase in the tangible priming case. Even then, only the collisions reduced, and driving time did not decrease.

**FIGURE 10:**
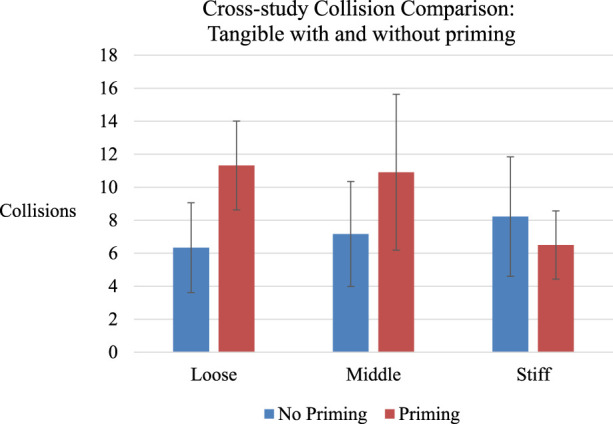
Collision results of the no priming (just joystick stiffness change) with the Tangible Priming condition. The no priming condition did not find a difference in collisions and has a trend moving in the opposite direction of the tangible case. The priming case appears to have cause more collisions in some cases. Error bars are 95% CI.

To investigate further, we looked in more detail at the specific collision results. First, we make a *post hoc* comparison between the collisions in our initial priming case (where priming reduced collisions) with the collisions in the follow-up no-priming study. Figure 10 highlights the no priming (blue) *versus* with-priming (red) studies, across the three tangible cases.

Surprisingly, the graph highlights that although collisions decreased based on the priming method within the priming study (the stiffer robot had fewer collisions), when comparing to the non-priming study (with near identical procedure) we can see that collisions were across-the-board lower or the same in the no-priming case than the priming case. That is, it appears as if operators performed better in general *without the priming*, and *worse with the priming*. We further considered the collision data from our descriptive study (Figure 9; note that the task was near identical and so comparison of collision counts is meaningful). Here, we see that the general number of collisions with descriptive priming (∼6.5–7.5) falls in line with the results in our no-priming tangible case, and are again lower than the tangible priming case. Given the *post hoc* and exploratory nature of this analysis, and confounds with comparing across studies, we do not feel it is appropriate or required to conduct statistical analyses on this point.

This leads to a new discussion regarding the impact of priming. We initially assumed that our priming improved operator behavior: within the tangible priming study we found that some priming types resulted in better operator performance than others. However, our meta-analysis highlights that–*in all cases–*the priming used either worsened or had no impact on performance. To complicate matters, Figure 10 still demonstrates how we cannot attribute the worse performance to usability (that some joystick stiffness settings were more difficult to control), as people performed better in the no-priming case even with the same joystick stiffness settings. The simplest explanation is that our tangible priming increased the number of collisions.

To consider this result, we re-visit the specific priming used. In this case, we told participants simply that the joystick stiffness reflected the robot’s “physical design.” We anticipated that the stiffness would reflect robot weight–where a stiff joystick would represent a heavy robot–but were unsure as to how. That is, we did not know whether participants would see this as more or less safe (heavy could mean slow or powerful, for example).

Why would this priming result in poorer performance? Perhaps priming led to higher cognitive load which could hinder driving. For example, perhaps people felt a looser joystick reflected a less well-controlled robot, which requires higher attention to drive properly. Another possibility is that our priming explanation, being somewhat vague (i.e., we did not tell participants exactly what stiffness meant), may have required participants to think deeply about what the robot was trying to communicate. In short, perhaps the priming did not affect the driving *per se*, but the fact that we had priming, may have caused participants to think more and thus have fewer cognitive resources to dedicate to driving. In the end, we cannot conclude why priming reduced performance instead of helped; this is a point that requires further study.

### 7.2 Why did perceptions of the robot change in the no priming case?

In order to remove the priming for the non-priming variant of the tangible study, instead of telling participants “each robot will interact with the joystick differently, based on the robot’s physical design,” we explicitly told them that the joystick stiffness simply changes, and that the robot itself does not change. While we intended this to remove the priming inherent in the earlier study design, participant perceptions still changed based only on the joystick stiffness.

To compare how the joystick stiffness impacts perceptions between the priming and no priming studies, we investigate the statistical ranks: these numbers represent the within-participant ordering of how participants rated the robot on the various measures (ranging from 1, first, to 3, third). Table 7 highlights a similar rating pattern between the priming and no priming studies: for example, for the loose joystick the robot was seen as faster, harder to steer, less durable, and less safe than the stiff robot in both cases. Thus, this suggests that the joystick stiffness itself–and perhaps not how we introduced the interface–shaped participant perceptions.

**TABLE 7:** The perceptual rankings of the tangible and no priming studies have been reproduced here for comparison.

	Tangible Priming			No Priming		
	Loose	Middle	Stiff	Loose	Middle	Stiff
Speed	2	2.4	1.7	2.1	2.2	1.7
Steering	1.6	2.2	2.2	1.5	2.4	2.1
Durability	1.7	2	2.3	1.6	2.2	2.2
Safety	1.7	2	2.3	1.6	2.4	2.0

Note that for No Priming, the speed measures were not found to be significant, though all other ratings here are.

One explanation is that, even though we asked participants to rate the robot’s performance, they may have rated the system as a whole (joystick, screen, networking quality, robot, etc.): a non-technical person may not understand how a joystick could separately impact control separate from the whole robot. If this is the case, the joystick stiffness may have had a similar impact on perceptions *of the whole system* in both studies. Alternatively, as the experimental design asks participants to compare and contrast the robots across the interfaces, there may have been pressure to find differences and participants may focus on the joystick as the only thing that changed between conditions.

However, we cannot attribute our findings simply to the pressure for participants to find differences. While we would expect this to result in noisy data (as people would just be guessing), Table 7 highlights a consistent ranking that emerged between participants–a ranking that was consistent with the priming study. Thus, it seems that some element of the joystick stiffness created a consistent impact on how people perceived the robot. To put it another way, the stiffness of the joystick may have primed participants, even when we explicitly tried to counter such an effect.

## 8 Implications for priming in human-robot interaction

Our initial motivation was to explore the use of priming as a simple and lightweight way for an interaction designer to shape how their interface is engaged and used. The summary results and meta-analysis from our three studies, however, highlights that the use and impacts of priming is not as simple as we initially conceived. In this section, we discuss the primary challenges we faced with employing priming, and present guidelines for investigating its use in human-robot interaction.

### 8.1 “To prime, or not to prime?” is not the right question

We attempted to conduct a study without priming by removing the earlier priming design and explicitly telling participants what we were doing. However, the resulting changes in operator perception of the robot still mirrored the earlier *with-priming* variant: this suggests that our attempt to conduct a study without the priming (removing it) not only failed, but the resulting study design had similar impacts as the with-priming design. In other words, we found that *priming may be unavoidable.*

To explain this seemingly contradictory result of priming while trying not to prime, we revisit the original goals of priming; the core idea is to shape and encourage people (through design) to draw from prior experiences to shape perceptions, expectations, and behavior. In the case of our no-priming experiment, *even though we told them the joystick stiffness has no bearing on robot function,* people would still experience that feeling and unconsciously draw from prior experiences relating to looser or tighter controls. In other words, the whole premise of removing priming may be flawed: we should always expect all designs, stimuli, and features to prime participants. The question then becomes *how* a design is priming people, not whether or not to employ priming.

On the one hand, this stance may seem obvious: design and human-computer interaction discusses at length the importance of what a design communicates to a user–e.g., see visibility of affordances (Gaver, 1991; Mcgrenere and Ho, 2000) and user-centered design in general (Beyer and Holtzblatt, 1997; Hassenzahl and Tractinsky, 2006; Brhel et al., 2015; Benyon, 2019). However, priming emphasizes that this effect goes far beyond physical design (e.g., a familiar shape, looks like it can be pushed, sat on, used as a bowl, *etc.*), and includes modifying perceived capabilities (e.g., speed) or tangible feel of a controller. The design of teleoperation interfaces is unfortunately often an afterthought in robotic systems (Rea et al., 2020; Rea and Seo, 2022), and our results emphasizes that due consideration should be given to smaller interface design cues that could prime. Robot system designers should be thinking broadly about the potential priming effects of all aspects of their interface design on user perceptions and behavior.

### 8.2 Subtleties of priming

The results from our studies highlight the subtleties and nuance of priming in interaction design, and the potential fallacy of trying to *remove* or *avoid* priming altogether. Given how many aspects of system design may impact how a person perceives and interacts with a robot, the goal then is to consider and be mindful of how priming may manifest and how to manage it. In reflecting on our work, we continue to find additional elements impacting the priming, and list some examples here.

One such example is that all of our priming methods relied on verbal descriptions to setup the priming; even the tangible priming was described to people. In some cases, we created an explicit connection (e.g., explaining the safe *versus* unsafe robot), while in others, we simply explained that a connection existed and left interpretation up to the participant. Even in our supposed “no-priming” case, we should expect this “no-priming” priming to impact performance as participants may remind themselves that they were told there was no difference. In all cases, the verbal description itself must be considered a part of the priming (in addition to, e.g., the infographic or tangible priming we employed), and many questions remain about its use. For example, how will an up-front verbal explanation impact people as time passes, how does the method of delivery (verbal vs. written), or tone of delivery, *etc.*, impact priming, and so forth.

Another unexpected result is the collateral effects of priming, such as potential increased cognitive load from the priming setup. For example, we discussed how it is possible that in informing participants of potential connections between variables may have increased mental load, where it required them to exert cognitive effort to constantly consider and assess the described connection. Future work is necessary to investigate how to leverage priming effects without such potential drawbacks.

### 8.3 Takeaways: Considerations for exploring priming for teleoperation

A key message of our paper is the simple fact that robot and interface designers need to consider priming in their teleoperation interface design, because of the wide range of factors that will impact what prior experience people draw from, and thus their perception of the robot and their resulting operation behavior. While we encourage the use of priming as a new (and relatively cheap) avenue for design, our studies highlight the effects are non-trivial to predict. However, we can provide both tools to brainstorm priming effects, as well as the following lessons about priming:

1) Priming can be leveraged by interface designers as a new tool for changing operator behavior and perception of the robot and its abilities.
2) Tangible and descriptive cues, without changing the robot, can change people’s perceptions of the robot’s abilities, even after extensive use.
3) Priming can impact perceptions of robot ability, general safety, driving experience, and product quality.
4) Priming can potentially change or improve operator behavior.
5) Priming can happen without intent, and can happen when explicitly trying to avoid priming effects, suggesting it may be impossible to avoid.

#### 8.3.1 Template for considering priming effects

Drawing from our experience, we provide the following brainstorming template for holistically considering priming in teleoperation. We emphasize that this is simply a summary of our own exploration presented in a succinct fashion and does not constitute grounded guidelines for employing priming in teleoperation. Instead, we envision that this may be a useful starting point for considering priming in a design.

1) Consider and enumerate all aspects of the teleoperation design:
  a) the overall system’s presentation*.* This includes packaging, product introductions, and any training or tutorials;
  b) the robot’s physical appearance, including robot morphology, sounds, and behaviors (e.g., has a manipulator? Tracks? Wheels?);
  c) the interface’s appearance and modalities (touch, haptics, sound, visual appearance), as well as the physical properties of those characteristics (update rate, resolution, response time, etc.);
  d) the environment the system will be used in, including the environment around the robot and the environment around the operator, such as noise, lighting, music, people, *etc.*
2) for each potential source of priming uncovered in #1, consider
  a) What existing systems or designs, whether real or fictional (e.g., in media), does this resemble? What prior experiences or memories may this trigger for people to shape their perceptions?
  b) What does this say about the overall product, company, and expected experience? E.g., does it suggest a rough prototype, a polished product, or expert system?
  c) Can this characteristic be modified to explicitly shape perceptions and behavior, e.g., by changing a color, shape, sound, key phrase, etc.,? Or, is it fixed due to external constraints (e.g., robot has tracks)?
  d) What secondary methods can be used to shape how this is perceived? Can a feature be described a certain way, or can it be compensated with complementary design (e.g., putting stylish stickers on a harsh metal frame)

Through this iterative brainstorming process of identifying potential sources of priming, and systematically considering how the source may impact perceptions, we envision that this approach can help teleoperation designers consider potential priming influences more systematically, and open design avenues that may have gone unexplored normally.

## 9 Limitations

While our priming methods were successful in changing participant perceptions of the robot and teleoperation experience, we only found teleoperation performance changes with the tangible method. We discussed potential reasons for those results above, but we note our quantitative measures in all three studies were not exhaustive; exploring other performance metrics (e.g. average robot velocity), will help us better understand the limits and potential of priming on teleoperation performance.

This work assumes that different people respond in similar ways to priming stimuli. However, it could be that different personalities may be more prone to risk taking, as suggested in transportation research (Jonah et al., 2001). In our results, our *safe* condition primed *safe* behavior, while some previous research suggests that the inverse may be true; for example, adding safety features to cars may result in less safe driving (Jonah et al., 2001). In teleoperation, a fast robot may encourage safer driving behavior from a cautious person, or a thrill-seeking operator may get excited and try push the robot to its limits. In fact, even how priming stimuli are interpreted in relation to safety could differ; for example a high top speed may be interpreted as fast and hard to control, or as a high quality robot with well-made components. We note that the science surrounding priming is still has conflicting results (Doyen et al., 2012), thus we recommend further inquiry into priming and teleoperation, considering a participant’s risk-tolerance.

Our scenario also limits the generalizability of our results. Our robot had a mostly steady but variable frame rate, though frame rate and latency can vary heavily in the real world and can have large effects on performance (Chen and Thropp, 2007; Jessie et al., 2007). Additionally, our obstacle course was designed to imitate a very crowded office or conference venue and make teleoperation difficult. However, environments with dynamic obstacles (such as people in a busy subway station), or wider spaces such as many museums will change the teleoperation experience. Additionally, our obstacle course was narrow, which may impact perception of speed and likely impact perceived safety compared to wider environments. As we noted earlier that research suggests that context is important for priming effects, investigating context for teleoperation and priming is an important consideration. We further noted this as a potential explanation for why our robot primed to be perceived as speed-limited did not perform well–its abilities may simply have not been suited to the task specifications. Priming our operators may have made them believe that one robot was more suited to the crowded obstacle courses, which explain perceptual or performance differences we observed.

Perhaps most importantly, we cannot speak to exactly *how* priming created the observed effects. For example, tangible priming may have created a sense of a large heavy robot, or a slow and easy to control robot, or a feeling of confidence, or another mechanism that steered them towards safer driving. Similarly, for perceptual measures, we cannot tell exactly what prior experiences participants drew upon due to our priming methods–we had our design motivation and intents, but we were not able to confirm those exact reasons (e.g., imagining a light, fragile, and fast sports car *versus* a slow and robust vehicle) were why the priming worked. Further work to understand the exact mechanisms of priming for teleoperation (i.e., what stimuli create what associations with what prior experiences) is needed.

### 9.1 Future work

Our results serve as a base to build from for future priming-based teleoperation interfaces. Even our two priming method labels–descriptive and tangible–are general and can be explored much further and much more deeply. For example, descriptive research may look at priming with actual demonstrations of robot behavior (using acting to prime the danger or ease of teleoperation), different robot morphologies, or different robot sounds. Further real-world case studies could be done observing how real user guides’ representations of robot capabilities could be affecting perception and use of the robots. Similarly, additional tangible methods could control force feedback effects such as adding shake to simulate rough terrain or a powerful motor. Exploring each technique in depth and starting to explore a broader range of priming techniques, is important for understanding the nuances of how priming can affect teleoperation.

We should also explore priming beyond portraying the robot as more or less safe. For example, sound could be used to prime different moods or atmospheres, or we could explore whether the enjoyment of teleoperating the robot could be primed. This is a new avenue to consider for teleoperation robot and interface design, and it leads to a broad range of future work.

Priming effects are often studied in the short term, such as our work in this paper. Long term effects of priming are less studied, and thus should be studied in the context of teleoperation; prior work suggests priming may last for hours or even months, even if new experiences contradict the priming (Sloman et al., 1988; Becker et al., 1997; Lindgaard et al., 2006). Perhaps short-term priming effects, especially when operators are first learning to drive a robot, may influence the development of safe long-term habits, but this must be formally studied. Such research would benefit both the psychology and teleoperation communities.

As discussed in our related work, the effectiveness of priming is still debated across scientific fields. As such, extensive replication and deeper and detailed experiment sets should be a priority to create more robust and replicable methods and results. We view our work as a small example of this–we tried to prime the same idea (safety) in multiple ways, and observed similar perceptual changes, giving us confidence in these results. Further replication is necessary to confirm our behavioral results, but we recommend all priming research endeavors to replicate their own or others’ findings due to the yet undecided nature of the field.

The mystery of how our no priming condition resulted in changes in perception of the identical robots people drove is also an important avenue to understanding priming. Part of the difficulty in pursuing this reason is our use of participant-volunteered responses; while we believe qualitative feedback is very important to understanding participant reactions to priming stimuli, it is inherently interpreted first by the participants themselves which makes it difficult to understand true causal relationships in priming. However, this made it difficult to determine the mechanisms by which our priming stimuli created our observed effects. We recommend future studies couple measures like we used with other, perhaps new techniques, to measure and understand a person’s internal thoughts, dialogue, and even subconscious processes when being primed. This would help understand priming at a deeper level, and give more insights and control over how to design for specific priming effects.

## 10 Conclusion

As teleoperated robots continue to develop and enter new environments and applications, operator error still remains a primary cause of critical incident. While we continue to improve robot algorithms and interface designs, in this work we highlight a new avenue for improving teleoperation that requires no changes to the robot or software: aiming to prime operators about the robot to shape their beliefs, and ultimately, their actions.

Through a series of experiments we demonstrated the potential of priming to shape operator beliefs. While in the end we failed to leverage priming to improve actual driving behavior (it only hindered in our cases), our work highlights that priming can impact behavior, and may have potential to improve it through future research. Our work highlights this potential avenue for further inquiry and exploration.

The ultimate result of this paper is simply that priming is unavoidable, nuanced, and will impact how operators perceive and use a robotic system. People draw from their prior experiences and knowledge to make decisions and shape their interactions with technologies. Thus, designers of teleoperation interfaces need to be acutely aware of this, and consider how their interface and robot designs impact people: what experiences will people draw from to decide how to use an interface? This paper provides a background on priming, and concrete experimental data, leading to implications and a brainstorming template, to assist interface designers in uncovering and considering potential priming effects in their work.

## Data Availability

The raw data supporting the conclusions of this article will be made available by the authors, without undue reservation.
